# Analysis of the phosphoproteome of CK2*α*^(–/–)^/Δ*α′* C2C12 myoblasts compared to the wild-type cells

**DOI:** 10.1098/rsob.220220

**Published:** 2023-02-22

**Authors:** Christian Borgo, Luca Cesaro, Tsuyoshi Hirota, Keiko Kuwata, Claudio D'Amore, Thomas Ruppert, Renata Blatnik, Mauro Salvi, Lorenzo A. Pinna

**Affiliations:** ^1^ Department of Biomedical Sciences, University of Padova, Padova 35122, Italy; ^2^ Institute of Transformative Bio-Molecules, Nagoya University, Nagoya 464-8601, Japan; ^3^ Zentrum für Molekulare Biologie der Universität Heidelberg (ZMBH), DKFZ-ZMBH Alliance, 69120 Heidelberg, Germany; ^4^ CNR Institute of Neurosciences, 35131 Padova, Italy

**Keywords:** protein kinase CK2, kinase inhibitors, cancer, phosphoproteomics

## Abstract

CK2 is a Ser/Thr protein kinase composed of two catalytic (*α*/*α*′) subunits and a non-catalytic β-subunit dimer, whose activity is often abnormally high in cancer cells. The concept that CK2 may be dispensable for cell survival has been challenged by the finding that viable CK2*α*/*α*′ knock-out myoblast clones still express small amounts of an N-terminally deleted *α*′ subunit generated during the CRISPR/Cas9 procedure. Here we show that, although the overall CK2 activity of these CK2*α*^(–/–)^/Δ*α*′ (KO) cells is less than 10% compared to wild-type (WT) cells, the number of phosphosites with the CK2 consensus is comparable to that of WT cells. A more in-depth analysis, however, reveals that the two phosphoproteomes are not superimposable according to a number of criteria, notably a functional analysis of the phosphoproteome found in the two types of cells, and variable sensitivity of the phosphosites to two structurally unrelated CK2 inhibitors. These data support the idea that a minimal CK2 activity, as in KO cells, is sufficient to perform basic housekeeping functions essential for cell survival, but not to accomplish several specialized tasks required upon cell differentiation and transformation. From this standpoint, a controlled downregulation of CK2 would represent a safe and valuable anti-cancer strategy.

## Graphical abstract

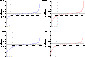

## 1. Introduction

Protein kinase CK2 (an abbreviation derived from the misnomer ‘casein kinase’-2) is one of the most pleiotropic members of the eukaryotic kinome, with hundreds of *in vitro* substrates known, and implicated in a plethora of cellular functions [1–8]. The CK2 holoenzyme, whose consensus sequence is specified by acidic residues downstream, the one at position *n* + 3 representing the minimum requirement, is a heterotetramer composed of two catalytic subunits (*α* and/or *α*′) bridged by a dimer of a non-catalytic *β* subunit whose regulatory roles are still incompletely understood [3].

A plethora of experimental data supports the implication of CK2 in many human diseases, with special reference to neoplasia [9–16]. In particular, the concept that many tumours rely on abnormally high CK2 expression/activity for their survival is corroborated by the observation that generally speaking the level of CK2 is higher in neoplastic cells as compared to their non-transformed counterparts [14,17,18] and are more susceptible to the cytotoxic effect of CK2 inhibitors [18]. Such suitability of CK2 as a broad anti-cancer target has been recently questioned by the observation that a newly developed CK2 inhibitor, SGC-CK2-1, which is more selective than those previously available, is poorly effective in reducing cell growth of many different tumour cell lines [19], thus challenging the concept that CK2 is indispensable for the viability of cancer cells.

‘Dispensability’ of CK2, however, is a complicated and thorny issue, influenced by the nature and developmental stage of the cell, and whose assessment cannot be based only on pharmacological approaches, which invariably ‘spare’ a substantial number of CK2 molecules that escape inhibition. Even the claim that CK2 may be dispensable for the viability of non-cancer cells, based on the generation of viable clones of C2C12 myoblasts genetically deprived of both CK2 catalytic subunits by the Crispr/Cas9 strategy [20], has been later challenged by the discovery that these cells still contain traces of a CK2*α*′ N-terminally deleted mutant (generated by the genetic manipulation) accounting for a tiny, yet detectable, CK2 activity presumably essential for cell viability [21].

We do not know if such a minimal CK2 activity would be sufficient to ensure the survival of cancer cells as well. But we suspect that this will not be the case, based on a number of clues including failure up to now to generate viable cancer cell clones deprived of both CK2 catalytic subunits by adopting the same strategy that proved successful in the case of the CK2*α*/*α*′^(−/−)^ myoblasts, and the accentuated cytotoxic response of cancer cells (as compared to non-cancer ones) to the reduction of endogenous CK2 [22–24]. A plausible scenario would be that a partial reduction of cellular CK2 might be still compatible with ‘normal’ cell viability by ensuring several basic functions, but not with the survival of transformed cells requiring additional functionalities which are no more afforded if CK2 drops under a critical level. This will reflect in the generation of distinct and only partially overlapping phosphoproteomes. To test this possibility we decided to perform a phosphoproteomics analysis of CK2*α*/*α*′^(−/−)^ myoblasts whose very low CK2 activity (8% of wild-type (WT)) is provided by a deleted form of CK2*α*′ [21] (CK2*α*^(−/−)^/Δ*α*′, (KO)), and to compare it with that of a previous study performed with their WT counterparts [25]. The results, presented in this report, will help to dissect CK2 functions variably required for cell survival.

## 2. Results

A list of the 2275 phosphosites quantified in KO myoblasts, whose only residual CK2 activity is due to the presence of an N-terminally deleted form of the *α*′ subunit [21] with a similar subcellular localization to the WT form (electronic supplementary material, figure S1), is provided in the electronic supplementary material, table S1. As shown in electronic supplementary material, figure S1, a similar distribution of the *β* subunit is present in the different subcellular fractions of both WT and KO cells.

As summarized in table 1, the majority of quantified phosphosites in KO cells displays the motifs recognized by Pro-directed protein kinases (44.6%) and by nearly all basophilic kinases (24.8%). A substantial number, however, (21.1%) conforms to the consensus of protein kinase CK2 (pS/pT-x-x-E/D/pS). As can be also seen in table 1, these numbers are not substantially altered with respect to those of WT cells (72.5% of quantified phosphopeptides are in common with those quantified in KO cells), where the proportion of phosphosites conforming to the CK2 consensus is slightly higher (24.7% versus 21.1%), while those of pS/pT-P and R-x-x-pS/pT phosphosites are a little decreased. Such a scenario was rather unexpected considering that in the KO cells CK2 activity is less than 10% of that of the WT [21]. Such a figure, measured with the aid of a specific peptide substrate [21], has been confirmed using casein as a substrate (see electronic supplementary material, figure S2), ruling out the possibility that it could be an artefact due to the usage of small peptide substrates.

**Table 1.** Sorting phosphosites quantified in KO and WT cells according to their phosphorylation motifs. Constructed with data drawn from the electronic supplementary material, table S1 and [25]. Phopshosites conforming or not to the commonest protein phosphorylation motifs [26] have been separately considered. Between brackets, the number of phosphosites conforming to a given consensus is expressed as a per cent of the total number. CK2 activity was calculated in [21].

	CK2 Activity (%)	*n* of sequences	[**pS/pT**]-x-x-[ED]	[**pS/pT**]-P	R-x-x[**pS/pT**]	other
*KO*	8	2275	481 (21.1%)	1014 (44.6%)	565 (24.8%)	541 (23.8%)
WT	100	2532	625 (24.7%)	1115 (44%)	581 (22.9%)	559 (22.1%)
WT & KO	—	1837	386	867	470	387

A possible explanation for the data of table 1 would be that the deleted form of the CK2*α*′ subunit, despite its paucity, instability and low activity, is still able to generate a substantial proportion of the CK2 phosphoproteome whose composition, intensity and distribution, however, may be altered as compared to WT. An alternative hypothesis would be that the phosphosites conforming to the CK2 consensus in these cells are generated by other kinases with similar or overlapping consensus.

To clarify this point whose general interest is not restricted to the specific case dealt with in this paper, we focused our attention on the actual implication of CK2Δ*α*′ in the generation of the phosphosites conforming to the CK2 consensus or at least to the majority of these.

To this aim, we first wanted to make sure that the contribution of CK2 to the generation of the phosphosites conforming to the CK2 consensus in the KO myoblasts is comparable to that observed in the WT cells [25]. To this end, advantage has been taken of two structurally unrelated specific CK2 inhibitors, CX4945 and GO289, whose narrow selectivity towards this kinase in WT C2C12 myoblasts has been previously assessed [25].

**Figure 1.**
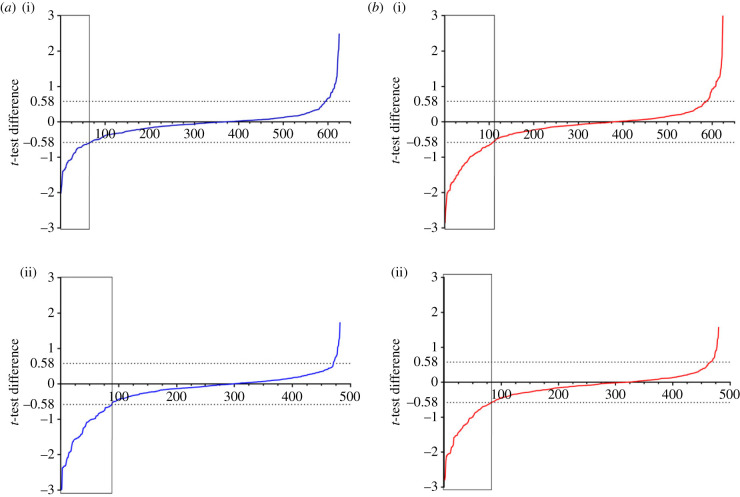
Distribution of the phosphorylation ratio of quantified phosphopeptides with CK2 consensus sequence. (*a*) Distribution of the phosphorylation ratio of phosphopeptides with CK2 consensus sequence (S/T-x-x-D/E/pS) quantified in WT cells (i) or KO cells (ii) treated with CX4945. The phosphosites that increase or decrease by more than 50% are highlighted by dashed lines (greater than 0.58 or less than 0.58). The vertical line parallel to the *y*-axis denoted the threshold (−0.58) between phosphosites that are decreased by less or more than 50% by treatment with inhibitors. (*b*) Distribution of the phosphorylation ratio of phosphopeptides with CK2 consensus sequence (S/T-x-x-D/E/pS) quantified in WT cells (i) or KO cells treated with GO289. The dashed line denotes the threshold between phosphopeptides that are decreased by more or less than 50%. The vertical line parallel to the *y*-axis denoted the threshold (−0.58) between phosphosites that are decreased by less or more than 50% by treatment with inhibitors.

A quantitative phosphoproteomics analysis on KO C2C12 cells either treated for 5 h with the CK2 inhibitors CX4945 (4 µM), GO289 (4 µM), or with vehicle (dimethylsulfoxide (DMSO)) has been performed using SILAC, as detailed in the material and methods section. In figure 1*a*, the distribution of the phosphorylation ratio of phosphopeptides conforming to the CK2 consensus quantified in either WT or KO both treated with the clinical grade CK2 inhibitor CX4945 is shown. Clearly, the efficacy of the inhibitor is significantly more pronounced towards the phosphosites generated in the KO cells 18% of which are greater than 50% decreased as compared to 10.4% decreased to the same proportion in the WT cells. This outcome is consistent with the concept that these phosphosites are indeed attributable to CK2 despite the paucity and the atypical nature of the CK2 activity expressed in these cells.

A similar result has been obtained by replacing CX4945 with another equally selective, structurally unrelated CK2 inhibitor, GO289 whose interactions with the CK2 catalytic domains are sharply different from those of CX4945 [27]. As shown in figure 1*b*, in this case, the two distribution curves are almost perfectly superimposable with a similar proportion of phosphosites greater than 50% decreased (16.8 versus 17.3%). A possible explanation could be that the CK2 catalytic machinery operating the KO cells is more sensitive to CX4945 than to GO289, at variance with what is observed in WT cells [25].

Such a conclusion has been confirmed by analysing a short list of KO phosphosites greater than 50% decreased by treatment with either CX4945 or GO289. These are on display in table 2. To note that the great majority of these phosphosites, conforms to the CK2 consensus, thus confirming that also in these cells genetically deprived of both WT CK2 catalytic subunits, the off-target effects of these two inhibitors are marginal as previously reported for WT cells [25]. If the same analysis is performed with phosphosites that are 50% increased rather than decreased by treatment with both inhibitors (electronic supplementary material, table S2), the outcome is sharply different in at least two respects: first the proportion of phosphosites increased is much smaller than that of phosphosites decreased (11 versus 67 with CX4945 and 9 versus 58 with GO289); second and more important while greater than 90% of the decreased phosphosites conform to the CK2 consensus, only 4 out of 20 increased phosphosites display this feature. These data are consistent with the view that while CK2 inhibitors promote a decrease of phosphosites conforming to the CK2 consensus by direct inhibition of the residual CK2 activity, the sporadically observed increase of a few phosphosites is the outcome of indirect processes ultimately impinging on kinases other than CK2.

**Table 2.** List of phosphosites quantified in KO cells decreasing more than 50% after inhibitors treatments. Phosphosites decreased by more than 50% in KO cells after (*a*) CX4945 treatment and (*b*) GO289 treatment. (*c*) Phosphosites present in both are shown in the bottom part of the table labelled as CX4945 & GO289. Italics denote phosphosites that do not conform to the CK2 consensus.

fold change	*t*-test difference	accession	name	gene	sequence
(*a*) CX4945					
1.53	−0.614	Q545R0	catenin alpha-1	Ctnna1	TPEELDD**S**DFETEDF
1.57	−0.648	Q3TXU8	membrane-associated progesterone receptor component 1	Pgrmc1	GEEPTVY**S**DDEEPKD
1.57	−0.655	Q9CT10	Ran-binding protein 3	Ranbp3	SPEAGED**S**DHEDGNY
1.58	−0.661	A2AMM0	muscle-related coiled-coil protein	Cavin4	FDPPIEL**S**SDEEYYV
1.58	−0.661	A2AMM0	muscle-related coiled-coil protein	Cavin4	DPPIELS**S**DEEYYVE
1.66	−0.729	Q61029-3	lamina-associated polypeptide 2, isoforms beta/delta/epsilon/gamma; lamina-associated polypeptide 2, isoforms alpha/zeta	Tmpo	*SDEEREP**T**PVLGSGA*
1.66	−0.734	P52479	ubiquitin carboxyl-terminal hydrolase 10	Usp10	LEDTGKG**S**EDEWEQV
1.68	−0.751	Q58E35	60S acidic ribosomal protein P1	Rplp1	KKEESEE**S**EDDMGFG
1.70	−0.769	B7ZC24	nuclear receptor coactivator 5	Ncoa5	*LLRSSAD**S**LPGELRG*
1.71	−0.776	Q8N7N5-2	DDB1- and CUL4-associated factor 8	Dcaf8	ASRDQDS**S**DDERALE
1.71	−0.776	B7ZC24	nuclear receptor coactivator 5	Ncoa5	RKERLLR**S**SADSLPG
1.74	−0.795	Q58E49	histone deacetylase; histone deacetylase 1	Hdac1	EDAIPEE**S**GDEDEED
1.78	−0.833	Q6P9Q6	FK506-binding protein 15	Fkbp15	DSQHCSL**S**GDEEDEL
1.85	−0.889	Q62018-3	RNA polymerase-associated protein CTR9 homologue	Ctr9	DEFVNDD**T**DDDLPVS
1.87	−0.902	Q9CQU1	microfibrillar-associated protein 1	Mfap1	PDYAPME**S**SDEEDEE
1.87	−0.902	Q9CQU1	microfibrillar-associated protein 1	Mfap1	DYAPMES**S**DEEDEEF
1.96	−0.971	Q64337	sequestosome-1	Sqstm1	QMESGNC**S**GGDDDWT
1.97	−0.979	Q8JZX4	splicing factor 45	Rbm17	SRRPDPD**S**DEDEDYE
1.97	−0.980	Q3UI57	DNA helicase; DNA replication licensing factor MCM3	Mcm3	KKASEDE**S**DLEDEEE
1.98	−0.988	Q9EP97	sentrin-specific protease 3	Senp3	PRPSFDA**S**ASEEEEE
2.00	−0.997	O54774	AP-3 complex subunit delta-1	Ap3d1	HSSLPTE**S**DEDIAPA
2.00	−0.999	Q91VM5	RNA-binding motif protein, X-linked-like-1; RNA-binding motif protein, X chromosome; RNA-binding motif protein, X chromosome, N-terminally processed	Rbmxl1	VEQATKP**S**FESGRRG
2.03	−1.024	Q6P542	ATP-binding cassette sub-family F member 1	Abcf1	KQLSVPA**S**DEEDEVP
2.06	−1.043	Q64511	DNA topoisomerase 2-beta	Top2b	VETINSD**S**DSEFGIP
2.07	−1.048	Q6P542	ATP-binding cassette sub-family F member 1	Abcf1	*MERLKQL**S**VPASDEE*
2.09	−1.064	Q91W67	ubiquitin-like protein 7	Ubl7	GFLFDGL**S**DDEDDFH
2.10	−1.073	P17095	high-mobility group protein HMG-I/HMG-Y	Hmga1	EGISQES**S**EEEQ___
2.11	−1.080	P17095	high-mobility group protein HMG-I/HMG-Y	Hmga1	EEGISQE**S**SEEEQ__
2.17	−1.116	Q8CH02	SURP and G-patch domain-containing protein 1	Sugp1	QHQHGYD**S**DEEVDSE
2.21	−1.144	E9Q5C9	nucleolar and coiled-body phosphoprotein 1	Nolc1	*VNSVKFD**S**E______*
2.26	−1.179	G5E8R4	serine/threonine-protein phosphatase 6 regulatory subunit 3	Ppp6r3	QQFDDGG**S**DEEDIWE
2.32	−1.213	A0A0J9YV20	protein SDA1 homologue	Sdad1	RKYLEID**S**DEESRGE
2.32	−1.215	H7BX26	centrosomal protein of 170 kDa	Cep170	*EDSKSIK**S**DVPVYLK*
2.37	−1.243	A2APD7	nucleolar protein 56	Nop56	SPKEEVA**S**EPEEAAS
2.38	−1.252	Q5SV64	myosin-10	Myh10	EGASLEL**S**DDDTESK
2.51	−1.327	E0CY39	SUN domain-containing protein 2	Sun2	*SSNMKHL**S**PAPQLGP*
2.53	−1.340	F7AL76	chromodomain-helicase-DNA-binding protein 8	Chd8	DDDLVEF**S**DLESEDD
2.68	−1.420	Q8VDD5	myosin-9	Myh9	RKGTGDC**S**DEEVDGK
2.68	−1.424	A0A0U1RPR0	eukaryotic translation initiation factor 3 subunit C	Eif3c	GKQPLLL**S**EDEEDTK
2.75	−1.461	Q3TM92	anaphase-promoting complex subunit 4	Anapc4	KIKEEVL**S**ESETEAH
2.78	−1.476	O54774	AP-3 complex subunit delta-1	Ap3d1	*KGKRRHS**S**LPTESDE*
2.79	−1.479	A0A140LIW6	DNA polymerase delta subunit 3	Pold3	RGKRVDL**S**DEEAKET
2.79	−1.481	Q8R2R3	alpha- and gamma-adaptin-binding protein p34	Aagab	DEIEGLS**S**DDEH___
2.86	−1.516	Q99KG3-3	RNA-binding protein 10	Rbm10	VAAYSGE**S**DSEEEQE
2.86	−1.516	Q99KG3-3	RNA-binding protein 10	Rbm10	AYSGESD**S**EEEQERG
2.88	−1.526	Q3ULL5	eukaryotic translation initiation factor 2 subunit 2	Eif2s2	______M**S**GDEMIFD
2.92	−1.547	Q9JMH9	unconventional myosin-XVIIIa	Myo18a	KNKLEGD**S**DVDSELE
2.96	−1.563	Q9Z0U1	tight junction protein ZO-2	Tjp2	DTRGSYG**S**DPEEEEY
3.00	−1.584	E9PUD0	zinc finger Ran-binding domain-containing protein 2	Zranb2	EYIEREE**S**DGEYDEF
3.09	−1.628	Q9DBR7-2	protein phosphatase 1 regulatory subunit 12A	Ppp1r12a	ENEQERQ**S**DTEDGSS
3.11	−1.637	Q05D44	eukaryotic translation initiation factor 5B	Eif5b	KSVPTVD**S**GNEDDDS
3.22	−1.687	P97310	DNA replication licensing factor MCM2	Mcm2	RRGLLYD**S**SEEDEER
3.50	−1.806	Q3UGC7	eukaryotic translation initiation factor 3 subunit J-A	Eif3j1	*AAAAAGD**S**DSWDADT*
3.74	−1.902	Q3TTV6	tuftelin-interacting protein 11	Tfip11	EEADSED**S**DAEEKPV
3.74	−1.902	Q3TTV6	tuftelin-interacting protein 11	Tfip11	*GAAEEAD**S**EDSDAEE*
3.75	−1.909	Q6A099	MKIAA0248 protein (fragment)	Gbf1	APDAGAQ**S**DSELPSY
3.92	−1.972	Q05D44	eukaryotic translation initiation factor 5B	Eif5b	PNSEAPL**S**GSEDADD
4.23	−2.080	F7AL76	chromodomain-helicase-DNA-binding protein 8	Chd8	VEFSDLE**S**EDDERPR
4.56	−2.188	A2A8V9	serine/arginine repetitive matrix protein 1	Srrm1	EPRKETE**S**EAEDDNL
5.01	−2.324	Q9CR29-2	coiled-coil domain-containing protein 43	Ccdc43	LAQYADV**T**DEEDEAD
5.01	−2.325	Q8K019-2	Bcl-2-associated transcription factor 1	Bclaf1	AKQKFHD**S**EGDDTEE
5.13	−2.360	E9Q616	AHNAK nucleoprotein (desmoyokin)	Ahnak	GHYEVTG**S**DDEAGKL
5.14	−2.361	A2APD7	nucleolar protein 56	Nop56	APKEELA**S**DLEEMAT
5.22	−2.384	Q99J36	THUMP domain-containing protein 1	Thumpd1	IDKDQQP**S**GSEGEDD
5.70	−2.511	Q80XU3	nuclear ubiquitous casein and cyclin-dependent kinase substrate 1	Nucks1	*DYSQFQE**S**DDADEDY*
7.77	−2.957	Q8K019-2	Bcl-2-associated transcription factor 1	Bclaf1	QEVLDYF**S**DKESAKQ
8.29	−3.051	Q6A099	MKIAA0248 protein (fragment)	Gbf1	SLDRGYT**S**DSEVYTD
(*b*) GO289					
1.52	−0.603	D3Z3M7	CAP-Gly domain-containing linker protein 1	Clip1	*SRYARKI**S**GTTALQE*
1.54	−0.627	Q61687	transcriptional regulator ATRX	Atrx	*REQEWDS**S**SDGTERL*
1.54	−0.627	Q61687	transcriptional regulator ATRX	Atrx	KREQEWD**S**SSDGTER
1.56	−0.638	Q6DFW4	nucleolar protein 58	Nop58	HIKEEPL**S**EEEPCTS
1.56	−0.639	Q9CQC5	Cdc42 effector protein 3	Cdc42ep3	*PVLKNAI**S**LPTIGGS*
1.62	−0.697	Q8CH02	SURP and G-patch domain-containing protein 1	Sugp1	QHQHGYD**S**DEEVDSE
1.62	−0.700	A2APD7	nucleolar protein 56	Nop56	SPKEEVA**S**EPEEAAS
1.67	−0.740	Q8N7N5-2	DDB1- and CUL4-associated factor 8	Dcaf8	ASRDQDS**S**DDERALE
1.68	−0.750	Q545R0	catenin alpha-1	Ctnna1	TPEELDD**S**DFETEDF
1.73	−0.792	Q3TXU8	membrane-associated progesterone receptor component 1	Pgrmc1	GEEPTVY**S**DDEEPKD
1.80	−0.848	Q8K2C9	very-long-chain (3R)−3-hydroxyacyl-CoA dehydratase 3	Hacd3	FDRWLDE**S**DAEMELR
1.83	−0.874	P52479	ubiquitin carboxyl-terminal hydrolase 10	Usp10	LEDTGKG**S**EDEWEQV
1.86	−0.892	Q58E35	60S acidic ribosomal protein P1	Rplp1	KKEESEE**S**EDDMGFG
1.91	−0.936	A0A0J9YV20	protein SDA1 homologue	Sdad1	RKYLEID**S**DEESRGE
1.99	−0.995	P17095	high-mobility group protein HMG-I/HMG-Y	Hmga1	EEGISQE**S**SEEEQ__
2.00	−1.000	Q6P9Q6	FK506-binding protein 15	Fkbp15	DSQHCSL**S**GDEEDEL
2.01	−1.004	H7BX26	centrosomal protein of 170 kDa	Cep170	*EDSKSIK**S**DVPVYLK*
2.01	−1.010	Q64337	sequestosome-1	Sqstm1	QMESGNC**S**GGDDDWT
2.03	−1.023	H7BX26	centrosomal protein of 170 kDa	Cep170	*RTEEDSK**S**IKSDVPV*
2.03	−1.024	Q6P542	ATP-binding cassette sub-family F member 1	Abcf1	KQLSVPA**S**DEEDEVP
2.03	−1.025	P17095	high-mobility group protein HMG-I/HMG-Y	Hmga1	EGISQES**S**EEEQ___
2.04	−1.025	Q91W67	ubiquitin-like protein 7	Ubl7	GFLFDGL**S**DDEDDFH
2.06	−1.045	E9Q5C9	nucleolar and coiled-body phosphoprotein 1	Nolc1	*VNSVKFD**S**E______*
2.15	−1.105	Q3UGC7	eukaryotic translation initiation factor 3 subunit J-A	Eif3j1	*AAAAAGD**S**DSWDADT*
2.19	−1.133	Q9JMH9	unconventional myosin-XVIIIa	Myo18a	KNKLEGD**S**DVDSELE
2.21	−1.144	Q6P542	ATP-binding cassette sub-family F member 1	Abcf1	*MERLKQL**S**VPASDEE*
2.23	−1.157	O54774	AP-3 complex subunit delta-1	Ap3d1	HSSLPTE**S**DEDIAPA
2.29	−1.193	G5E8R4	serine/threonine-protein phosphatase 6 regulatory subunit 3	Ppp6r3	QQFDDGG**S**DEEDIWE
2.30	−1.202	Q99KG3-3	RNA-binding protein 10	Rbm10	VAAYSGE**S**DSEEEQE
2.30	−1.202	Q99KG3-3	RNA-binding protein 10	Rbm10	AYSGESD**S**EEEQERG
2.35	−1.235	A0A140LIW6	DNA polymerase delta subunit 3	Pold3	RGKRVDL**S**DEEAKET
2.54	−1.347	E9PUD0	zinc finger Ran-binding domain-containing protein 2	Zranb2	EYIEREE**S**DGEYDEF
2.57	−1.360	Q3TTV6	tuftelin-interacting protein 11	Tfip11	EEADSED**S**DAEEKPV
2.57	−1.360	Q3TTV6	tuftelin-interacting protein 11	Tfip11	*GAAEEAD**S**EDSDAEE*
2.62	−1.391	Q9JIK5	nucleolar RNA helicase 2	Ddx21	SDAPGEE**S**SSETEKE
2.63	−1.393	Q9DBR7-2	protein phosphatase 1 regulatory subunit 12A	Ppp1r12a	ENEQERQ**S**DTEDGSS
2.71	−1.437	A0A0U1RPR0	eukaryotic translation initiation factor 3 subunit C	Eif3c	GKQPLLL**S**EDEEDTK
2.87	−1.523	Q3ULL5	eukaryotic translation initiation factor 2 subunit 2	Eif2s2	______M**S**GDEMIFD
2.88	−1.525	Q9Z0U1	tight junction protein ZO-2	Tjp2	DTRGSYG**S**DPEEEEY
2.88	−1.525	A2A8V9	serine/arginine repetitive matrix protein 1	Srrm1	EPRKETE**S**EAEDDNL
2.95	−1.562	Q8VDD5	myosin-9	Myh9	RKGTGDC**S**DEEVDGK
2.98	−1.575	Q9EQC8	papillary renal cell carcinoma (translocation-associated)	Prcc	LQKGDSD**S**EEDEPAK
3.00	−1.586	O54774	AP-3 complex subunit delta-1	Ap3d1	RRHSSLP**T**ESDEDIA
3.07	−1.618	O54774	AP-3 complex subunit delta-1	Ap3d1	*KGKRRHS**S**LPTESDE*
3.46	−1.793	Q8K019-2	Bcl-2-associated transcription factor 1	Bclaf1	AKQKFHD**S**EGDDTEE
3.48	−1.798	Q80XU3	nuclear ubiquitous casein and cyclin-dependent kinase substrate 1	Nucks1	*DYSQFQE**S**DDADEDY*
3.57	−1.834	A2APD7	nucleolar protein 56	Nop56	APKEELA**S**DLEEMAT
3.61	−1.851	Q05D44	eukaryotic translation initiation factor 5B	Eif5b	KSVPTVD**S**GNEDDDS
3.63	−1.858	Q05D44	eukaryotic translation initiation factor 5B	Eif5b	PNSEAPL**S**GSEDADD
4.11	−2.039	Q9DBG7	signal recognition particle receptor subunit alpha	Srpra	QLQDLDC**S**SSDDEGA
4.11	−2.039	Q9DBG7	signal recognition particle receptor subunit alpha	Srpra	LQDLDCS**S**SDDEGAT
4.11	−2.041	Q99J36	THUMP domain-containing protein 1	Thumpd1	IDKDQQP**S**GSEGEDD
4.17	−2.061	Q6A099	MKIAA0248 protein (fragment)	Gbf1	APDAGAQ**S**DSELPSY
4.18	−2.064	Q569Z6	thyroid hormone receptor-associated protein 3	Thrap3	KWAHDKF**S**GEEGEIE
4.31	−2.109	F7AL76	chromodomain-helicase-DNA-binding protein 8	Chd8	VEFSDLE**S**EDDERPR
4.82	−2.268	Q8K019-2	Bcl-2-associated transcription factor 1	Bclaf1	QEVLDYF**S**DKESAKQ
6.44	−2.686	E9Q616	AHNAK nucleoprotein (desmoyokin)	Ahnak	GHYEVTG**S**DDEAGKL
6.99	−2.805	Q6A099	MKIAA0248 protein (fragment)	Gbf1	SLDRGYT**S**DSEVYTD
(*c*) CX4945 and GO289					
2.17	1.62	Q8CH02	SURP and G-patch domain-containing protein 1	Sugp1	QHQHGYD**S**DEEVDSE
3.57	1.62	A2APD7	nucleolar protein 56	Nop56	SPKEEVA**S**EPEEAAS
1.71	1.67	Q8N7N5-2	DDB1- and CUL4-associated factor 8	Dcaf8	ASRDQDS**S**DDERALE
1.53	1.68	Q545R0	catenin alpha-1	Ctnna1	TPEELDD**S**DFETEDF
1.57	1.73	Q3TXU8	membrane-associated progesterone receptor component 1	Pgrmc1	GEEPTVY**S**DDEEPKD
1.66	1.83	P52479	ubiquitin carboxyl-terminal hydrolase 10	Usp10	LEDTGKG**S**EDEWEQV
1.68	1.86	Q58E35	60S acidic ribosomal protein P1	Rplp1	KKEESEE**S**EDDMGFG
2.32	1.91	A0A0J9YV20	protein SDA1 homologue	Sdad1	RKYLEID**S**DEESRGE
2.10	1.99	P17095	high-mobility group protein HMG-I/HMG-Y	Hmga1	EEGISQE**S**SEEEQ__
1.78	2.00	Q6P9Q6	FK506-binding protein 15	Fkbp15	DSQHCSL**S**GDEEDEL
2.32	2.01	H7BX26	centrosomal protein of 170 kDa	Cep170	*EDSKSIK**S**DVPVYLK*
1.96	2.01	Q64337	sequestosome-1	Sqstm1	QMESGNC**S**GGDDDWT
2.03	2.03	Q6P542	ATP-binding cassette sub-family F member 1	Abcf1	KQLSVPA**S**DEEDEVP
2.11	2.03	P17095	high-mobility group protein HMG-I/HMG-Y	Hmga1	EGISQES**S**EEEQ___
2.09	2.04	Q91W67	ubiquitin-like protein 7	Ubl7	GFLFDGL**S**DDEDDFH
2.21	2.06	E9Q5C9	nucleolar and coiled-body phosphoprotein 1	Nolc1	*VNSVKFD**S**E______*
3.50	2.15	Q3UGC7	eukaryotic translation initiation factor 3 subunit J-A	Eif3j1	*AAAAAGD**S**DSWDADT*
2.92	2.19	Q9JMH9	unconventional myosin-XVIIIa	Myo18a	KNKLEGD**S**DVDSELE
2.07	2.21	Q6P542	ATP-binding cassette sub-family F member 1	Abcf1	*MERLKQL**S**VPASDEE*
2.00	2.23	O54774	AP-3 complex subunit delta-1	Ap3d1	HSSLPTE**S**DEDIAPA
2.26	2.29	G5E8R4	serine/threonine-protein phosphatase 6 regulatory subunit 3	Ppp6r3	QQFDDGG**S**DEEDIWE
2.86	2.30	Q99KG3-3	RNA-binding protein 10	Rbm10	AYSGESD**S**EEEQERG
2.86	2.30	Q99KG3-3	RNA-binding protein 10	Rbm10	VAAYSGE**S**DSEEEQE
2.79	2.35	A0A140LIW6	DNA polymerase delta subunit 3	Pold3	RGKRVDL**S**DEEAKET
3.00	2.54	E9PUD0	zinc finger Ran-binding domain-containing protein 2	Zranb2	EYIEREE**S**DGEYDEF
3.74	2.57	Q3TTV6	tuftelin-interacting protein 11	Tfip11	*GAAEEAD**S**EDSDAEE*
3.74	2.57	Q3TTV6	tuftelin-interacting protein 11	Tfip11	EEADSED**S**DAEEKPV
3.09	2.63	Q9DBR7-2	protein phosphatase 1 regulatory subunit 12A	Ppp1r12a	ENEQERQ**S**DTEDGSS
2.68	2.71	A0A0U1RPR0	eukaryotic translation initiation factor 3 subunit C	Eif3c	GKQPLLL**S**EDEEDTK
2.88	2.87	Q3ULL5	eukaryotic translation initiation factor 2 subunit 2	Eif2s2	______M**S**GDEMIFD
2.96	2.88	Q9Z0U1	tight junction protein ZO-2	Tjp2	DTRGSYG**S**DPEEEEY
4.56	2.88	A2A8V9	serine/arginine repetitive matrix protein 1	Srrm1	EPRKETE**S**EAEDDNL
2.68	2.95	Q8VDD5	myosin-9	Myh9	RKGTGDC**S**DEEVDGK
2.78	3.07	O54774	AP-3 complex subunit delta-1	Ap3d1	*KGKRRHS**S**LPTESDE*
5.01	3.46	Q8K019-2	Bcl-2-associated transcription factor 1	Bclaf1	AKQKFHD**S**EGDDTEE
5.70	3.48	Q80XU3	nuclear ubiquitous casein and cyclin-dependent kinase substrate 1	Nucks1	*DYSQFQE**S**DDADEDY*
5.14	3.57	A2APD7	nucleolar protein 56	Nop56	APKEELA**S**DLEEMAT
3.11	3.61	Q05D44	eukaryotic translation initiation factor 5B	Eif5b	KSVPTVD**S**GNEDDDS
3.92	3.63	Q05D44	eukaryotic translation initiation factor 5B	Eif5b	PNSEAPL**S**GSEDADD
5.22	4.11	Q99J36	THUMP domain-containing protein 1	Thumpd1	IDKDQQP**S**GSEGEDD
3.75	4.17	Q6A099	MKIAA0248 protein (fragment)	Gbf1	APDAGAQ**S**DSELPSY
4.23	4.31	F7AL76	chromodomain-helicase-DNA-binding protein 8	Chd8	VEFSDLE**S**EDDERPR
7.77	4.82	Q8K019-2	Bcl-2-associated transcription factor 1	Bclaf1	QEVLDYF**S**DKESAKQ
5.13	6.44	E9Q616	AHNAK nucleoprotein (desmoyokin)	Ahnak	GHYEVTG**S**DDEAGKL
8.29	6.99	Q6A099	MKIAA0248 protein (fragment)	Gbf1	SLDRGYT**S**DSEVYTD

**Figure 2.**
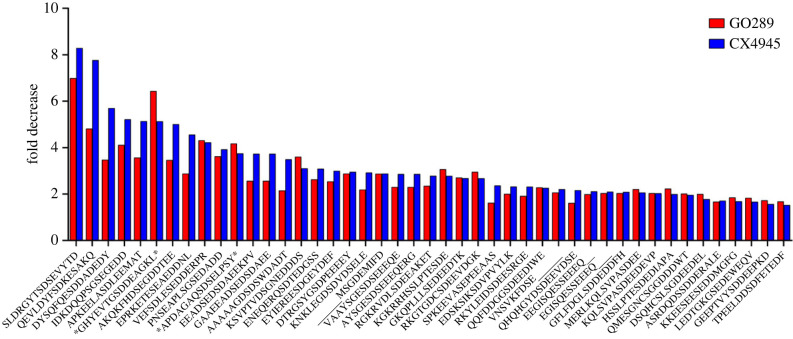
The decrease of phosphopeptides upon treatment with either CX4945 or GO289 of KO cells. The graph compares the effects of CX4945 or GO289 on phosphosites that have been quantified in CX4945 and GO289 treated cells and that are decreased by more than 50%. Phosphosites that are in common with that found in [25] are denoted by asterisks.

Comparative scrutiny of parts (a) and (b) in table 2 reveals that 45 phosphosites (extrapolated in table 2*a*) have been quantified among those decreased greater than 50% by both CX4945 (67% of those in table 2*a*) and GO289 (77% of those in table 2*c*). As shown in figure 2 in nearly all these cases the decrease promoted by CX4945 is larger than that caused by GO289, corroborating the view that these phosphosites are more sensitive to the former inhibitor. Interestingly this outcome was the opposite if the same experiment was run with the WT cells [25]: in that experiment, the phosphosites with CK2 consensus decreased greater than 50% by both CX4945 and GO289 were 26 and all of them were invariably more sensitive to GO289 than to CX4945 [25]. This is due at least in part to the different susceptibility of CK2 WT and KO to the two inhibitors as disclosed by experiments where their efficacy was tested on the whole CK2 activity detectable in the lysates of either WT or KO cells (electronic supplementary material, figure S3). It should be also noted that in both cases the relative efficacy of the two inhibitors deeply varies according to the nature of the substrate considered.

A general lesson that can be drawn from the above experiments is that the in-cell efficacy of kinase inhibitors may be variable depending on the nature of the substrate considered, thus warning against the usage of individual targets as ‘reporters’ of kinase activity to draw general conclusions about the expectable overall effects of a given inhibitor.

The experiments of electronic supplementary material, figure S3 have been also performed in the presence of a third CK2 inhibitor, SGC-CK2-1, recently described as the first-in-class in terms of selectivity [19] The data shown in the electronic supplementary material, figure S3 are noteworthy in at least four respects: first they show that the efficacy of SGC-CK2-1 as CK2 inhibitor is quite comparable to that of CX4945 both in WT and in KO cells; second they show that CX4945 and SGC-CK2-1 are less potent than GO289 as inhibitors of WT CK2, while the opposite applies to KO; third the close superimposition of the CX4945 and SGC-CK2-1 curves in both panels suggests that phosphoproteomic analyses performed with the latter would disclose a scenario similar to that observed with CX4945 rather than with GO289; last but not least, considering that CX4945 and GO289 display identical potency towards the two catalytic subunits of CK2 [27] one has to assume that the sharp differences between WT and KO cells outlined in the electronic supplementary material, figure S3 cannot be uniquely accounted for by the lack of *α* in the KO cells but must also depend on the altered targeting and/or catalytic properties of the deleted form of the α′ subunit as compared to its WT form. Apparently the N-terminal deletion increases susceptibility to both CX4945 and SGC-CK2-1, while impairing inhibitability by GO289 (electronic supplementary material, figure S3, compare panels A and B).

**Figure 3.**
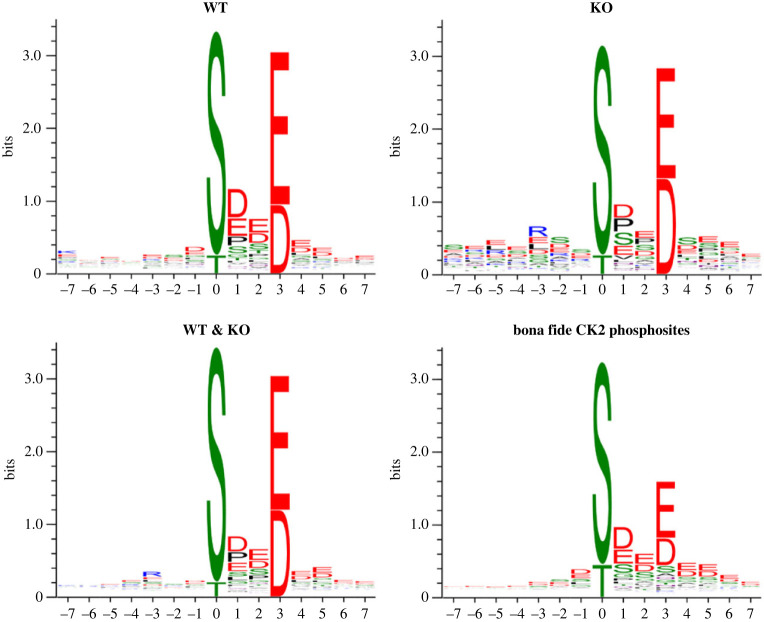
WebLogo analyses. The WebLogos obtained from all phosphosites quantified only in WT cells from [25], only in CK2*α*^(−/−)^/Δ*α*′ (KO) cells, and of phosphosites quantified in both WT and KO cells (WT & KO) are shown and compared to the WebLogo of *bona fide* CK2 phosphosites drawn from PhosphoSitePlus (www.phosphosite.org).

Next, we wanted to investigate if there are significant functional differences between the phosphoproteomes conforming to the CK2 consensus generated in the KO cells as compared to the WT cells. To this aim, the phosphosites conforming to the CK2 consensus quantified either in both WT and KO cells (386 altogether) or uniquely in one or another of the two kinds of cells (239 and 95, respectively) have been separately considered. As shown in figure 3, their WebLogos are almost identical conforming also to the WebLogo of *bona fide* CK2 targets. It may be worthy to note however that a distinctive feature of KO cells WebLogo is a significant positive selection of Pro and Arg at positions *n* + 1 and *n* − 3, respectively, which are both negative determinants for CK2 [1] but required as essential positive determinants by Pro-directed and many basophilic kinases, respectively [28]. As discussed below, this could be symptomatic of a contribution of kinases other than CK2 to the generation in the KO cells of phosphosites conforming to the CK2 consensus.

**Figure 4.**
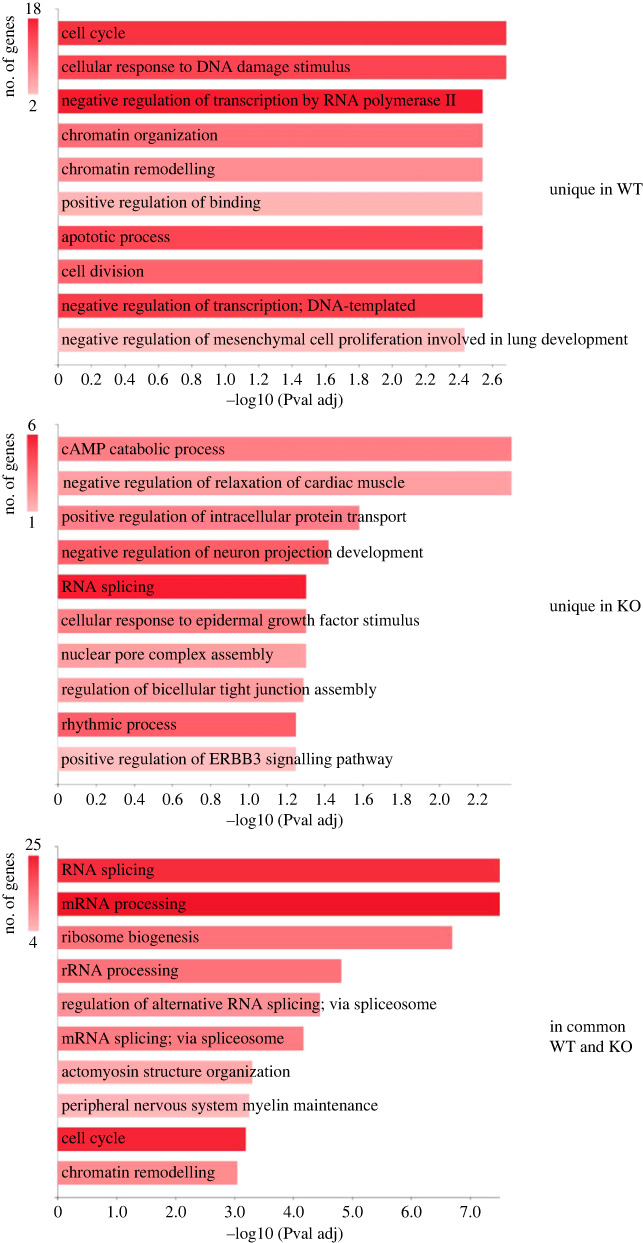
Gene ontology analyses. Functional analysis of proteins containing phosphosites quantified only in WT cells, only in KO cells, or in both cell lines assigned using GeneCoDis3 webserver [29]. The size of the annotation bar (*x*-axes) is proportional to −log10 (*p*-value adj.).

Functional analysis of the proteins including these sites, however, (figure 4) reveals significant differences regarding a number of functions, notably cellular response to DNA damage stimulus, chromatin organization and remodelling among those predominant for proteins quantified only on WT cells which are instead scarcely or not represented at all in the knocked-out cells. By contrast, some functions, in particular those related to RNA processing, are predominant among proteins whose CK2 phosphosites have been quantified also in the KO cells (figure 4).

**Figure 5.**
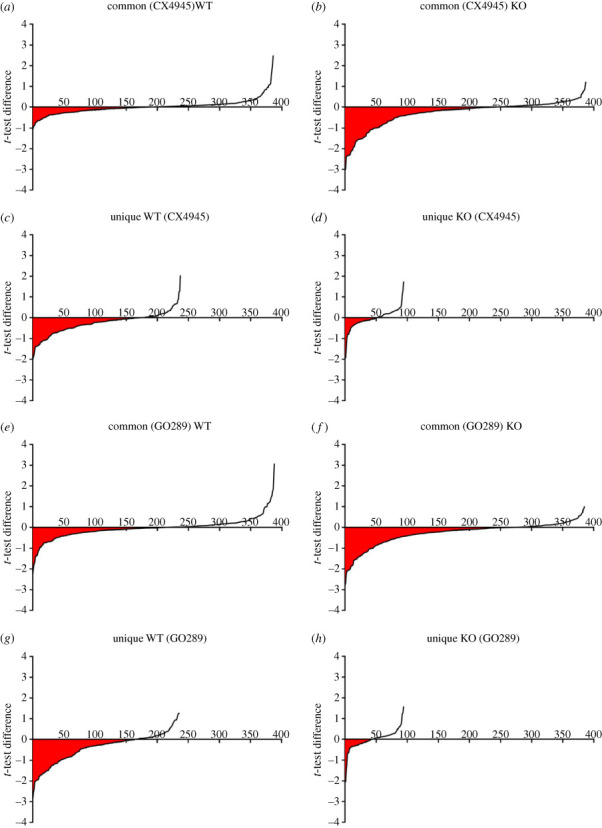
Distribution of the phosphorylation ratio of different subsets of quantified phosphopeptides with CK2 consensus following CX4945 or GO289 treatment. (*a–d*) Distribution of the phosphorylation ratio of phosphopeptides with CK2 consensus sequence (pS/pT-x-x-D/E/pS) following CX4945 treatment quantified in common between (*a*,*b*) WT and KO cells or (*c,d*) only in WT and KO cells. (*e*–*h*) Distribution of the phosphorylation ratio of phosphopeptides with CK2 consensus sequence (pS/pT-x-x-D/E/pS) following GO289 treatment quantified in common between (*e*,*f*) WT and KO cells or (*g,h*) only in WT and KO cells. The red areas highlighted below the abscissa of the plots of this figure reflect the responsiveness of different subsets of CK2 phosphosites to cell treatment with the inhibitor, their sizes being determined both by the number of decreased phosphosites (measured in the abscissa) and their extent of inhibition, as indicated on the negative *y*-axis. Data referring to WT cells are drawn from [25].

Further insight into this important issue was provided by analysing separately the different subsets of CK2 phosphosites quantified in cells treated with CK2 inhibitors whose distribution of the phosphorylation ratio is shown in figure 5. The red areas highlighted below the abscissa of the plots of this figure reflect the responsiveness of different subsets of CK2 phosphosites to 5 h cell treatment with the inhibitor, their sizes being determined both by the number of decreased phosphosites (measured in the abscissa) and their extent of inhibition, as indicated on the negative *y*-axis.

If the phosphosites in common for both WT and KO cells are considered, it appears that their responsiveness to CX4945 treatment is much more pronounced in KO cells (electronic supplementary material, figure S3, compare panels A and B), consistent with the notion that these cells display a CK2 activity more than 10-fold less than WT (table 1; electronic supplementary material, figure S2). In a way, this was also expectable on the basis of figure 5 denoting an overall larger decrease of phosphosites conforming to the CK2 consensus in the KO as compared to the WT cells.

Interestingly, however, the responsiveness to CX4945 of CK2 phosphosites that have been quantified only in WT cells (figure 5*c*) is much more pronounced than that displayed in the same cells by phosphosites that are in common also in KO cells (figure 5*a*). Since in this case, CK2 activity in WT cells is the same one possible explanation is that ‘unique’ phosphosites, typical of WT cells but not of KO cells, undergo, on average, a faster turnover.

Intriguingly the opposite applies to phosphosites exclusively quantified in KO cells but not in WT cells (figure 5*d*): these are quite refractory to CX4945 treatment as compared to KO cells phosphosites that are also quantified in WT cells (figure 5*b*).

A similar scenario is observed if another CK2 inhibitor, GO289, structurally unrelated to CX4945, is employed (figure 5*e–h*), supporting the view that the overall outcome is not significantly biased by the off-target effects of the CK2 inhibitors used. Rather the especially modest efficacy of both CK2 inhibitors on the phosphorylation level of phosphosites quantified only in KO cells may suggest that some of these phosphosites might be generated by kinases other than CK2 despite they conform to the CK2 consensus.

## 3. Discussion

In a 1999 paper entitled ‘CK2 a protein kinase of the next millennium’, the Nobel Laureate Edwin G. Krebs wrote that the title was intended ‘to emphasize the fact that CK2 is such a rich topic for investigation that research involving this enzyme will continue for decades to come’ [30]. Such a complexity of CK2 biology reflects not only in its terrific pleiotropy [1,5] but even more in its implication in such a wide variety of cell functions that situations where CK2 might be unnecessary are hardly conceivable. This underlies the postulate of CK2 indispensability.

Given this scenario, the generation by the Crispr/Cas9 methodology of viable C2C12 myoblasts entirely deprived of both CK2 catalytic subunits [20] came as a striking surprise and promoted a revisitation of CK2 pleiotropy based on the presumption that phosphosites conforming to the CK2 consensus in these knock-out cells might be due to other protein kinases [31].

Later on, however, an unexpected discovery was made that during the genetic manipulation performed to knock-out both catalytic subunits, *α* and *α*′, a mutant of the *α*′ subunit was generated which despite its deletion of 8 AA close to the N-terminal and very low-expression level still exhibits detectable catalytic activity amounting to 8% as compared to the WT cells [21]. Such a serendipitous event on one side discloses the possibility that the phosphosites conforming to the CK2 consensus in the KO cells are at least in part generated by the residual deleted form of CK2*α*′, on the other provides a tool to dissect CK2 functions required just for survival from more specialized CK2 functions that may be lost in the KO cells. Our present work sheds light on both issues.

Firstly in fact our data show that the phosphoproteome of C2C12 myoblasts where both the catalytic subunits of protein kinase CK2 have been knocked out is still composed of a substantial proportion of phosphosites genuinely generated by CK2.

Such a conclusion has been made possible by the usage of two specific, structurally unrelated CK2 inhibitors, CX4945 and GO289. Here we show that on average the sensitivity of the phosphosites conforming to the CK2 consensus to both inhibitors in the KO cells is comparable to that observed in the WT cells. This finding, in conjunction with the observation that nearly all the phosphosites greater than 50% reduced by treatment of the KO cells with both inhibitors do conform to the CK2 consensus, strongly supports the conclusion that most of these phosphosites are indeed generated also in the KO cells by the residual CK2 activity attributable to the CK2Δ*α*′ form present in them.

Pertinent to this would be the question as to whether the phosphoproteomic alterations observed in the KO cells are simply attributable to the lack in these cells of the CK2*α* subunit and the presence of reduced amounts of the *α*′ subunit or is instead also the consequence of a targeting perturbation caused by the N-terminal deletion of the residual CK2*α*′ subunit. It is well known in fact that the two catalytic subunits of CK2 display partially different functions probably also reflecting in different targeting [32–34]. A thorough answer would require a comparative phosphoproteomic analysis of CK2*α*^(−/−)^ cells where only CK2*α*′ survives. Presently available data are consistent with a scenario whereby the presence of the N-terminally deleted CK2*α*′ subunit in KO cells displays an altered targeting in addition to a reduced sensitivity to the inhibitor GO289 as outlined in electronic supplementary material, figure S3. Especially telling are in this respect the gene ontology analyses of figure 4 and even more the quantification in KO cells of a subset of phosphosites conforming to the CK2 consensus which were not found in WT cells, as discussed below. An alternative explanation could be that the CK2*α*^(−/−)^/Δ*α*' cells express very low amounts of *β* subunit, whose association with the catalytic subunits represents a well-known mechanism by which CK2 substrate specificity can be modulated [3,35]. This possibility, however, would be hardly consistent with the observation that the KO cells express sufficient amount of *β* (electronic supplementary material, figure S1) able to associate with the deleted form of the *α*′ subunit [21].

Our present study also provides additional pieces of information, which can contribute to a better understanding of the multifarious cellular functions of CK2, as detailed below.

The majority of the CK2 phosphosites quantified in the WT cells were also detected in the KO cells consistent with the notion that the residual activity due to the CK2Δ*α*′ deleted mutant is sufficient to generate a large proportion of the CK2-dependent phosphoproteome, albeit presumably phosphorylated to a lower level. It may be worthy to note in this respect that a large number of these phosphosites were also quantified in a previous study [31] among those drastically reduced upon knocking out of both catalytic subunits, consistent with the concept that in these cells the phosphoproteome generated by the CK2Δ*α*′ mutant on the average is phosphorylated to a lesser extent. This in turn may account for the observation that in the KO cells the CK2 phosphosites tend to be more readily responsive to CK2 inhibition.

A substantial minority of the phosphosites conforming to the CK2 consensus (about 40%) could be uniquely quantified in the WT cells and not in the KO cells. On average these phosphosites display a markedly higher susceptibility to CK2 inhibitors as compared to phosphosites quantified both in WT and in the KO cells reflecting the concept that their turnover is faster leading to their substantial dephosphorylation during the 5 h treatment with the inhibitors. Making the reasonable assumption that the CK2 inhibitors do not affect the activity of the phosphatase(s) responsible for the dephosphorylation of the CK2 sites we can conclude that these sites are not significantly phosphorylated in the CK2*α*^(−/−)^/cell due to the minimal level of CK2 activity present in these cells.

The concept that the phosphoproteomes generated by CK2 in the WT cells and in cells expressing only the CK2Δ*α*′ mutant reflect the implication of CK2 in partially different functions is also supported by two additional criteria: firstly a gene ontology analysis revealing significantly different patterns of biological processes affected by proteins whose phosphosites belong to the two categories; second the opposite efficacy of the two inhibitors on the phosphosites generated by CK2 in the WT and the KO cells, with GO289 more potent than CX4945 on the former [25], while CX4945 is more potent than GO289 in the latter ones (figure 2). Such a behaviour of CK2*α*^(−/−)^/Δ*α'* cells is attributable to conformational alterations of the *α*′ subunit causing an increased sensitivity to CX4945 and SGC-CK2-1, and decreased susceptibility to GO289 (electronic supplementary material, figure S3). These conformational changes are also presumably responsible for altered targeting. Structural studies will be necessary to shed light on the molecular features underlying the peculiar properties of this CK2*α*′ mutant.

The genesis and significance of the phosphosites conforming to the CK2 consensus quantified uniquely in the KO cells remain an open question. By sharp contrast with the phosphosites shared with the WT cells, these are especially refractory to both CK2 inhibitors, raising the suspicion that many of them may be not generated by CK2. This may be true of those phosphosites which also include the consensus for proline-directed and/or basophilic kinases, as disclosed by their WebLogos (figure 3) revealing a positive selection of Pro and Arg at +1 and –3, respectively. Such an explanation, however, hardly applies to the majority of these phosphosites whose local features are those of typical CK2 sites. Another possible explanation could be that the CK2Δ*α*′ mutant artificially generated in the KO cells performs a number of ‘misphosphorylations’ not susceptible to be readily reversed by dedicated protein phosphatases and therefore refractory to 5 h cell treatment with CK2 inhibitors. Further investigation will be necessary to clarify this point.

## 4. Conclusion

For sake of simplicity, we can group the phosphosites conforming to the CK2 consensus detected in the course of our work into three categories: (i) Those which were detected only in the WT cells but not the KO ones, displaying on average a marked responsiveness to CK2 inhibitors denoting their fast turnover rate. These are likely to be implicated in specialized functions requiring normal to high CK2 cellular level and/or the integrity of the heterotetrameric CK2 holoenzyme. These functions, partially outlined by the gene ontology analyses of figure 4, are presumably lost in the KO cells. (ii) Phosphosites detected both in the WT and KO cells, which in these latter tend to be more prone to dephosphorylation upon blockage of CK2, consistent with their lower phosphorylation level. Expectedly these phosphosites are implicated in basal functions required for cell survival and accounting for the viability of the KO cells; (iii) Phosphosites which were detected only in the KO cells but not in the WT ones. These are especially refractory to cell treatment with CK2 inhibitors, suggesting that they might be generated by kinases other than CK2. Failure to detect them in WT cells, however, remains hard to explain unless assuming that in the background of cells where the catalytic subunits of CK2 have been knocked-out and replaced by the generation of the CK2*α*′ subunit deletion mutant a complex genetic rearrangement takes place giving rise either to the expression/activation of novel kinase/s) or to the turning off of the expression/activation of complementary phosphatases which in WT cells maintain these sites in a predominantly dephosphorylated form. In any case, these phosphosites reveal a situation where the N-terminal deletion mutant of CK2*α*′ is causative of cellular perturbations hardly expectable in CK2*α*^(−/−)^ cells expressing only the CK2*α*′ subunit.

More generally speaking we can conclude that the serendipitous generation of an N-terminally deleted form of CK2*α*′ in C2C12 myoblasts where both the catalytic subunits of CK2 had been knocked-out has provided a model useful for discriminating between CK2 functions indispensable for cell viability [36], which are preserved in the knocked-out cells thanks to the CK2Δ*α*′ residual activity (less than 10% compared to WT) and more specialized functions which are lost in these cells because they require higher CK2 activity and/or the integrity of both catalytic subunits. Such a functional dissection reflects in the generation of distinct phosphoproteomes which are partially overlapping and whose detailed analysis will provide a tool for deciphering how ‘a constitutively active kinase like CK2 can be a participant in critical cellular processes*’* [36], a major issue in the field of signalling via protein phosphorylation [3].

## 5. Opening up

The progress of knowledge is not always a linear process dictated by a rational experimental design and the unambiguous interpretation of its outcome. Quite often serendipity perturbs such an ‘ideal’ scenario with unanticipated ‘accidents’ whose underlying explanation escapes the understanding capability of investigators. These serendipitous accidents may generate frustration, disappointment and sometimes embarrassment in scientists. A frequent reaction is to disregard these mishaps by simply dropping thorny topics and turning to more rewarding ones. Often, however, this will later turn out to be a big mistake because serendipitous artefacts may reflect physiologically relevant events and provide unanticipated tools for the unravelling of complicated issues.

An example is provided by the data presented in this paper, exploiting a serendipitous accident to dissect the functionalities of one of the most pleiotropic protein kinases. In our case, the unanticipated event was the generation in C2C12 myoblasts in which both catalytic subunits of protein kinase CK2 had been successfully knocked-out by the Crispr/Cas9 methodology of the deletion mutant of one of the two subunits whose catalytic activity, albeit drastically reduced, was still sufficient to ensure cell survival. For a while this mutant remained undetected, giving rise to the wrong feeling that cell viability does not require CK2 and that phosphosites conforming to the CK2 consensus are generated in the KO cells by kinases other than CK2. The discovery of the mutant has provided a different explanation of the experimental data, conferring to the KO cells the potential of a model useful to shed light on the multifarious functions of this highly pleiotropic kinase. Available data show that the phosphoproteomes generated by CK2 in WT versus KO cells are only partially overlapping, thus allowing to discriminate between phosphosites implicated in basal functions required for the survival of both WT and KO cells, whose phosphorylation takes place in both kinds of cells and phosphosites whose phosphorylation is detectable only in WT cells, presumably requiring normal to high CK2 activity, implicated in more specialized functions which are defective in the KO cells. An in-depth analysis of these different subsets of the CK2 phosphoproteomes will pave the way towards a better understanding of the biology of this pleiotropic protein kinase.

## 6. Materials and methods

### 6.1. Materials

Protease inhibitor cocktail was from Calbiochem (Darmstadt, Germany), while phosphatase inhibitor cocktails 2 and 3 were from Sigma-Aldrich (Dorset, UK). CX-4945 (5-[(3-Chlorophenyl)amino]-benzo[c]-2,6-naphthyridine-8-carboxylic) was purchased from Glixx Laboratories (South Borough, MA, USA). GO289 was synthesized as described in [27]. SGC-CK2-1 was purchased from MedChemExpress (NJ, USA) Solutions were made in DMSO. Labelled amino acids for SILAC experiments were purchased from Silantes GmbH (Muchen, Germany). Unlabelled amino acids (L-arginine, L-lysine, L-glutamine and L-proline) were purchased from Sigma. Anti-CK2*α*′ (sc-514403) and anti-LDH (sc-133123), anti-RPS6 (sc-74459) were from Santa Cruz Biotechnology (Dallas, TX, USA). Anti-p84N5 (ab487) was from Abcam (Cambridge, UK).

### 6.2. Cell culture

Mouse myoblast C2C12 cells were maintained in 5% CO_2_ in DMEM supplemented with 10% FBS, 2 mM L-glutamine, 100 U ml^–1^ penicillin and 100 mM streptomycin, in an atmosphere containing 5% CO_2_.

Cells were regularly passed and used for experiments within one month after thawing (10–15 passages). C2C12 KO cells have been generated in [20]. The KO clone used in this study presents an homozygous deletion of 8 AA close to the N-terminus [21].

### 6.3. Stable isotope labelling and cell lysis

Cells were grown in DMEM containing either unlabelled l-arginine and l-lysine (Arg^0^, Lys^0^) (light) or equimolar amounts of l-[^13^C_6_]-arginine and L-Lysine-^2^H_4_ (Arg^6^, Lys^4^) (medium) or l-[^13^C_6_,^15^N_4_]-arginine and l-[^13^C_6_,^15^N_2_]-lysine (Arg^10^, Lys^8^) (heavy) supplemented with 200 mg l^–1^ light proline to prevent the conversion of arginine to proline ^44^, 2 mM L-glutamine, 1% penicillin/streptomycin and 10% dialysed Fetal Bovine Serum (Silantes GmbH). Cells were grown in SILAC medium for eight cell doublings and treated as indicated. Three biological replicates with a label-swap strategy were performed for each of the triplex SILAC experiments (sample 1: DMSO, light; CX4945, medium; GO289, heavy; sample 2: CX4945, light; GO289, medium; DMSO, heavy; sample 3: GO289, light; DMSO, medium; CX4945, heavy).

Labelled cells were solubilized by the addition of ice-cold buffer containing 20 mM HEPES (pH 8.0), 9 M urea, protease inhibitors EDTA-free (1 tablet/10 ml, Roche) and phosphatases inhibitors Cocktail 2 and 3 (Sigma) and sonicated. Cell debris was removed by centrifugation and SILAC-encoded samples were pooled at a ratio of 1 : 1 : 1.

Proteins (0.3 mg/300 µl) were reduced with 25 mM tris(2-carboxyethyl)phosphine (TCEP) at 37°C for 15 min, alkylated using 25 mM iodoacetamide at 37°C for 30 min in the dark, both with gentle shaking (1000 rpm) following the standard protocol. Proteins were digested with Lys-C (FUJIFILM Wako) at an enzyme-protein ratio of 1 : 100 at 37°C for 3 h. This Lys-C digest was diluted to 2 M urea with 100 mM Tris-HCl, pH 8.5, followed by digestion with trypsin (Promega) at an enzyme-protein ratio of 1 : 100 at 37°C overnight. After digestion was stopped by adding 1/20 volume of 20% TFA, digested peptides were desalted by a MonoSpin C18 spin column (GL Science) according to the manufacturer's instruction. Eluates were evaporated *in vacuo* to dryness, reconstituted in 200 µl of 80% acetonitrile (0.1% TFA), and subjected to phosphopeptide enrichment using Fe(III)-IMAC cartridges (5 µl) on a AssayMAP Bravo platform (Agilent) at a flow rate of 5 µl min^–1^ according to manufacturer's protocol. Phosphopeptides were eluted three times with 20 µl of 20% acetonitrile (1% TFA), 20 µl of 25% acetonitrile (5% ammonia) and 25% acetonitrile (5% pyrrolidine), respectively. The second- and third-eluted fraction were mixed and acidified with 20% TFA. The eluted fraction was concentrated and desalted using GL-Tip SDB (GL Science) according to the manufacturer's instructions. The desalted sample was dried down and stored at −80°C until subjected to LC-MS/MS. The first eluent and the second/third eluents were separately analysed by LC-MS/MS.

### 6.4. LC-MS/MS and data analysis

Samples were analysed by nano-flow reverse-phase liquid chromatography followed by tandem MS, using a Q-Exactive hybrid mass spectrometer (Thermo). A capillary reverse-phase HPLC-MS/MS system was composed of a Dionex U3000 gradient pump equipped with VICI CHEMINERT valve, and Q-Exactive was equipped with a nano-electrospray ionization source (AMR, Japan). The desalted peptides were loaded into a separation capillary C18 reverse-phase column (NTCC-360/100–3–125, 125 × 0.1 mM, Nikkyo Technos, Japan). Xcalibur 3.0.63 system (Thermo) was used to record peptide spectra over the mass range of *m/z* 350–1800. Repeatedly, MS spectra were recorded followed by the generation of 10 data-dependent high-energy collisional dissociation MS/MS spectra from 10 highest intensity precursor ions. Multiple charged peptides were chosen for MS/MS experiments because of their good fragmentation characteristics. Raw files have been analysed by Max Quant [37] and statistical analysis has been performed with Perseus [38].

WebLogos were generated using WebLogo3 (http://weblogo.threeplusone.com/) [39,40].

Bioinformatic analyses for the functional characterization of proteins were performed with Genecodis [29].

### 6.5. CK2 kinase activity assay

Peptide kinase activity: 2 µg of lysate proteins were incubated for 10 min at 30°C in 25 µl of a phosphorylation medium containing 50 mM Tris-HCl (pH 7.5), 100 mM NaCl, 10 mM MgCl_2_, 3 µM Staurosporine, 400 µM peptide substrate RRRADDSDDDDD (R_3_AD_2_SD_5_) and 20 µM [*γ*-^33^P]ATP (1000 cpm pmol^−1^). Assays were stopped by absorption onto phosphocellulose p81 filters (PerkinElmer, Waltham, MA, USA), which were washed four times in 75 mM phosphoric acid and analysed by a Scintillation Counter (PerkinElmer).

Protein kinase activity: 2 µg of lysate proteins were incubated for 10 min at 30°C in 25 μl of a phosphorylation medium containing 50 mM Tris-HCl (pH 7.5), 100 mM NaCl, 10 mM MgCl_2_, 3 µM Staurosporine, 1 µg *β*-casein (Sigma-Aldrich) and 20 µM [*γ*-^33^P]ATP (1000 cpm pmol^−1^). Assays were stopped by adding Laemmli buffer and samples were subjected to 13% SDS-PAGE. Radioactive ^33^P- *β*-casein was evidenced by analysing the dried gel with a Cyclone Plus Storage PhosphorSystem (PerkinElmer).

### 6.6. Subcellular fractionation of C2C12 cells

WT or KO C2C12 cells were detached and washed twice with PBS. Cells (5 × 10^6^) were resuspended in 300 µl of ice-cold hypotonic buffer containing 20 mM Tris-HCl, pH 7.8, 1% (v/v) Nonidet P-40, and protease and phosphatase inhibitor cocktails. After 30 min, samples were passed through a 23 G needle 80 times using a 1 ml syringe. Samples were immediately centrifuged at 800 × g for 10 min at 4°C. Cytoplasmic fractions were collected as supernatants, while the pellets containing the nuclear fractions were extracted with 150 µl of lysis buffer containing 20 mM Tris-HCl (pH 7.5), 1% Triton X-100, 10% glycerol, 1 mM EDTA, 150 mM NaCl and protease and phosphatase inhibitor cocktails. Cytoplasmic fractions were further centrifuged at 1 00 000 × g for 1 h to precipitate the membraneous fraction. Membraneous fractions have been lysed with 100 µl with the same buffer used for nuclear fraction. Equal amounts of protein were loaded on 12% SDS-PAGE, blotted on Immobilon-P membranes (Millipore), processed by western blot with the indicated antibody and detected by chemiluminescence on ImageQuant LAS 500 (GE Healthcare Life Sciences).

## Data Availability

The data are provided in the electronic supplementary material [41].
